# MicroRNA-202 suppresses glycolysis of pancreatic cancer by targeting hexokinase 2

**DOI:** 10.7150/jca.43379

**Published:** 2021-01-01

**Authors:** Shuang-Jia Wang, Xiu-Dong Li, Lu-Peng Wu, Ping Guo, Liu-Xing Feng, Bin Li

**Affiliations:** Department of Hepato-Biliary-Pancreatic and Vascular Surgery, The First Affiliated Hospital of Xiamen University, Xiamen, Fujian 361003, P.R. China

**Keywords:** miR-202, Hexokinase 2, Glycolysis, Pancreatic cancer

## Abstract

**Purpose:** Various studies have identified miR-202 critically participated in the development of different cancers. However, the potential mechanisms underlying the carcinogenesis of pancreatic cancer (PC) still remains elusive.

**Methods:** In the study, cell proliferation assay, colony formation assay, EdU incorporation assay, Luciferase reporter assay, lactate production, glucose consumption assay, real-time PCR and western blot were used to investigate the mechanism of hexokinase 2 (HK2) regulated by miR-202 in pancreatic cancer in vitro and in vivo.

**Results:** Here we found that miR-202 was decreased in the PC tissues, and its low expression was correlated with a poor prognosis of PC patients. Overexpression of miR-202 in PC cells reduced cell proliferation and tumorigenesis by impairing glycolysis, while downregulation of miR-202 promoted the cells proliferative capacity. Mechanically, we demonstrated that HK2, an enzyme that catalyzes the irreversible rate-limiting step of glycolysis, as the direct target of miR-202. Overexpression of miR-202 suppressed both the mRNA and protein levels of HK2, whereas re-introduction of HK2 abrogated miR-202-mediated glycolytic inhibition. In addition, the expression of miR-202 was negatively associated with HK2 level in a cohort of PC tissues.

**Conclusion:** Our findings validate the mechanism that miR-202 reprograms the metabolic process to promote PC progression, thus providing potential prognostic predictors for PC patients.

## Introduction

Pancreatic cancer is one of the most common causes of cancer-related deaths in the world, with a poor 5-year survival rate 1. It's known with insidious symptoms, rapid deterioration and early metastasis 2. Despite the progress of cancer screen and diagnosis, the incidence of PC is still increasing both in China and the United States 3, 4. Due to occult symptoms, lack of effective treatments and deferred diagnosis, the prognosis of PC remains very poor 1. Therefore, it is urgent to make further investigation and seek a better way to control its morbidity and mortality.

MicroRNAs (miRNAs) are small non-coding RNA molecules, comprised of 18-23 nucleotides (nt) that are involved in various biological behaviors by binding to the 3'-untranslated regions (3'UTRs) of target genes 5, 6. Several miRNAs have been reported as a tumor suppressor or oncogene in the regulation of cell proliferation, metastasis, apoptosis, and senescence, etc. in PC 7, 8. Previous reports showed that miR-202 could function as a tumor suppressor in non-small cell lung cancer 9, gastric cancer 10 and cervical cancer 11. However, the role of miR-202 in PC is controversial. The previous study showed that overexpression of miR-202 inhibited apoptosis in PANC-1 cells^12^. However, a recent study showed that miR-202 attenuated TGFβ1-Induced EMT in PC 13. Accordingly, identifying the role of miR-202 in the pathogenesis of PC is crucial for the diagnosis and treatment of PC.

In the present study, we found that miR-202 was down-regulated in PC tissues and cell lines. By gain-of-function and loss-of-function experiments, we found miR-202 impaired pancreatic cancer glycolysis and subsequently repressed cell proliferation by directly targeting hexokinase 2 (HK2). Our results suggest that miR-202 functions as a tumor suppressor and may be a potential therapeutic target for PC therapy.

## Material and methods

### Ethics

The present study conformed to the ethical guidelines of the World Medical Association Declaration of Helsinki-Ethical Principles for Medical Research Involving Human Subjects and was approved by the Institutional Research Board at the First Affiliated Hospital of Xiamen University, China. Written informed consent was obtained from each patient. Institutional Ethics Committee approval for this project was provided before the commencement of the study. All specimens were handled according to ethical and legal standards.

### Cell lines and cell cultures

Human PC cell lines (AsPC-1, BxPC-3, SW1990, PANC-1, HS766t) and a non-malignant pancreatic epithelial cell hTERT-HPNE were purchased from the Cell Resource Center, Shanghai Institute of Biochemistry and Cell Bank at the Chinese Academy of Sciences, May 2016. Cells were propagated in RPMI-1640 medium supplemented with 10% fetal bovine serum under a humidified incubator at 37C containing 5% CO2.

### Reagents and plasmid transfection

miR-202 mimic and relevant negative control was purchased from GenePharma (Shanghai, China), and transfected into cells as reported previously.^12^ Cells were harvested for further experiment post-transfection 48 hours. HK2(sc-374091) and GAPDH(sc-47724) antibody was purchased from Santa Cruz Biotechnology(CA, USA). To set up the stable transfection system of anti-miR-202, anti-miR-202 sequence were cloned from miRZip-202 construct (System Biosciences) and inserted into pSilencer4.1 system. For the stable expression of miR-202, pMSCV-miR-202 was generated by cloning the genomic pre-miR-202 gene into backbone plasmid pMSCV. The stable cells were selected by puromycin post 48h transfection.

### Cell proliferation assay and colony formation assays

Cell proliferation was performed by the Cell Counting Kit-8 kit (CCK-8) (cat#C0038, Beyotime, Hangzhou, China). Indicted cells were plated onto 96-well plates (4×10^3^ cells per well) and further incubated for 1-5 day. Next, the absorbance at 450 nm was measured with a microplate reader (Thermo Scientific, Waltham, MA, USA). For the colony formation assay, 1 × 10^3^ cells were plated in each well of a 10-cm dish and incubated at 37 °C for 2 weeks. The cells were fixed with 4% paraformaldehyde and stained with 1% crystal violet. Cell colonies were counted and analyzed.

### EdU incorporation assay

Cells were incubated with EdU (5-Ethynyl-2′-deoxyuridine) at a concentration of 10μM for 1.5 h and analyzed using a Click-iT® EdU Alexa Fluor® Imaging Kit (cat#C10337, Molecular Probes, USA) according to the manufacturer's protocols. Then the EdU-positive cells were calculated and analyzed.

### RNA extraction and quantitative qRT-PCR

Total RNA was extracted from tissues and cells using RNeasy Mini kit (Qiagen) and measured using a Nano Drop ND-1000 spectrophotometer (Nano Drop Technologies, Rockland, DE, USA) according to the manufacturer's instructions. The miRNA sequence-specific primer and probes for miR-202, endogenous control RNU6B and TaqMan 2X PCR Master mix for TaqMan miRNA assays were purchased from Applied Biosystems. The real-time PCR assay was performed on an ABI PRISM7500 system (Applied Biosystems, USA). Data analysis was performed with ΔCt values of miRNAs from each sample were calculated by normalizing with internal control U6 and each value were mean of 3 replicates. The following primers were used: miR-202, forward 5′-CCTCCCAGGCTCACGAGGCT-3′ and reverse 5′-GGTGCAGGTGCACTGGTGCA-3′; U6 forward: CTCGCTTCGGCAGCACA, U6 reverse: AACGCTTCACGAATTTGCGT.

### Western Blotting

Whole cell or tissue extracts were prepared using RIPA buffer. The protein samples were separated on 10% SDS-PAGE and then transferred to a PVDF membrane. The membrane was blocked with 5% non-fat milk incubated with indicated primary antibodies. Protein bands were visualized by ECL Detection Reagent (Pierce). GAPDH was used as an internal control.

### Luciferase reporter assay

The wild-type 3'UTR of HK2 containing the putative miR-202 binding site was amplified by PCR then cloned into the downstream of the luciferase gene in the pGL3 vector (Promega, Madison, WI, USA). The mutations of the miR-202 putative binding sites in HK2 3'UTR were also synthesized by PCR. *Renilla* reporter pRL-TK and indicated plasmids (PGL3-WT-HK2, PGL3-Mut-HK2) were co-transfected into cells. Forty-eight hours after transfection, cells were lysed, and luciferase activity was assessed using the dual-luciferase reporter assay system (Promega). All experiments were repeated at least 3 times.

### Lactate production and glucose consumption

Cells were cultured in RPMI without phenol red for 15 h, and the supernatants were collected for measurement of lactate or glucose concentrations. Lactate levels were measured using the Lactate Assay kit (Bio-Vision, Mountain View, USA) and glucose levels were quantified with using a glucose assay kit (Sigma-Aldrich). All values were normalized to total protein levels using the Pierce BCA Protein assay (Thermo Scientific).

### Cellular glucose-6-phosphate and ATP levels

The cellular levels of glucose-6-phosphate and ATP were measured using a Glucose-6-phosphate Fluorometric Assay kit (Cayman, Michigan, USA) and a CellTiter-Glo Luminescent Cell Viability Assay (Promega) according to the manufacturer's instructions.

### Clinical specimens

Pancreatic tumor and adjacent noncancerous tissues were obtained from 101 pancreatic cancer patients receiving pancreatoduodenectomy at the Department of Hepato-Biliary-Pancreatic and Vascular Surgery of the First Affiliated Hospital of Xiamen University from January 2009 to December 2012. The clinicopathological data including age, sex, tumor size, CA-199 level, pathological grade, vascular invasion, lymph node metastasis, and TNM, were collected from patient files (Table 1). These patients included 48 males and 53 females, with a median age of 62 years (range, 38-79 years). These patients were followed-up closely until December 31, 2017.

### Animal xenograft model

PANC-1 cells (1×10^7^) that stably expressed indicated plasmids were harvested and subcutaneously injected into the lower back of 6-week-old male nude mice (each group 8 mice). Tumor growth was monitored by caliper measurements. Excised tumors were weighed at indicated days and sacrificed after 4 weeks and examined for the growth of subcutaneous tumors. All the experimental procedures involving animals were conducted in accordance with Institutional Animal Care guidelines.

### Statistical Analysis

Statistical significance of differences between groups was assessed using the GraphPad Prism6 software. Student's t-test or one-way ANOVA was applied to determine the significance between groups. Statistical analyses between different treatments, in different cell cohorts or at different time points were performed using two-way ANOVA with the Bonferroni's correction. The overall survival and disease-free survival were estimated according to the Kaplan-Meier method and significance was determined by the log-rank test. Pearson correlation analysis was used to determine the expression of miR-202 and HK2 of PC patients. All experiments were repeated at least 3 times. Statistical significance was concluded at *P < 0.05, **P < 0.01, ***P < 0.001; # represents no statistical significance.

## Results

### miR-202 is downregulated in PC and negatively associated with prognosis

**Figure 1:**
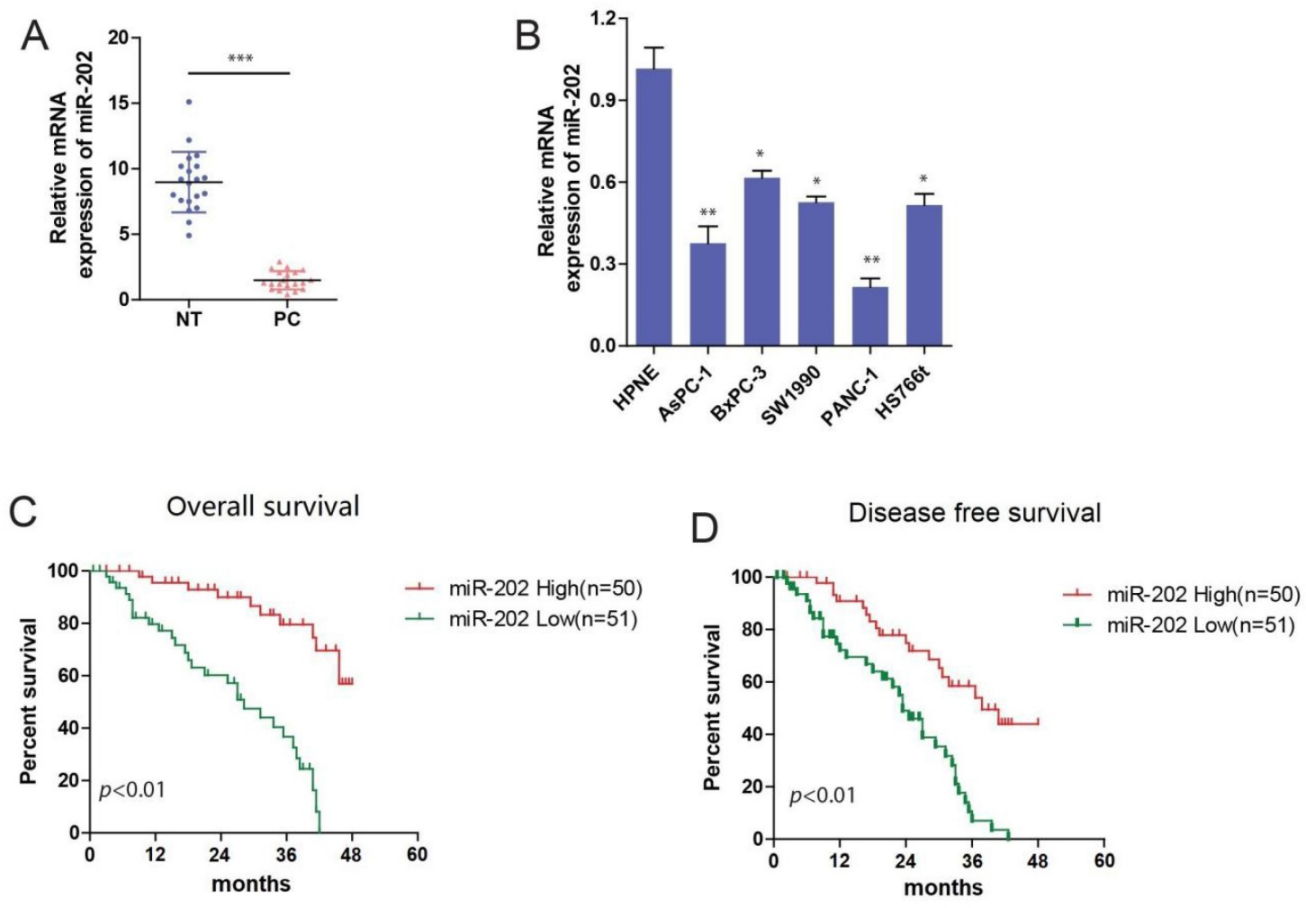
** miR-202 is decreased in PC and associated with poor prognosis.** (A) qRT-PCR analysis of miR-202 expression in human PC tissue samples and adjacent normal tissues from 20 PC patients. (B) qRT-PCR analysis of miR-202 expression in human PC cell lines and noncancerous human pancreas epithelial cells. Kaplan-Meier curves of overall survival (C) and disease-free survival (D) for PC patients with high/low miR-202 expression.

To identify the role of miR-202 in PC carcinogenesis, we investigated the expression of miR-202 in PC tissues and adjacent normal tissues by using qRT-PCR. We observed that miR-202 expression was significantly decreased in PC tissue compared with adjacent normal tissues (Figure 1A). Next, we used qRT-PCR to detect the endogenous expression levels of miR-202 in PC cell lines including AsPC-1, BxPC-3, SW1990, PANC-1, HS766t, and a non-malignant pancreatic epithelial cell hTERT-HPNE. Compared to normal epithelial cells, the expression of miR-202 was obviously decreased in PC cells. (Figure 1B). We further determined the overall survival (OS) and disease-free survival (DFS) by Kaplan-Meier analysis. We found that the OS and DFS of patients with high miR-202 expression were significantly longer compared with low miR-202 expression (Figure 1C and 1D). Additionally, analysis between clinicopathologic features and miR-202 expression levels in PC cases revealed that miR-202 expression was negatively correlated with tumor size and lymph node metastasis (Table **1**). The multivariate analysis revealed that tumor size, TNM stage, and miR-202 expression had a significant prognostic influence on OS (Table **2**). These results suggest that the miR-202 level is downregulated in PC tissues and cell lines and that it is negatively correlated with the prognosis of PC patients.

### miR-202 impairs PC cell growth and tumorigenicity *in vitro* and *in vivo*

**Figure 2:**
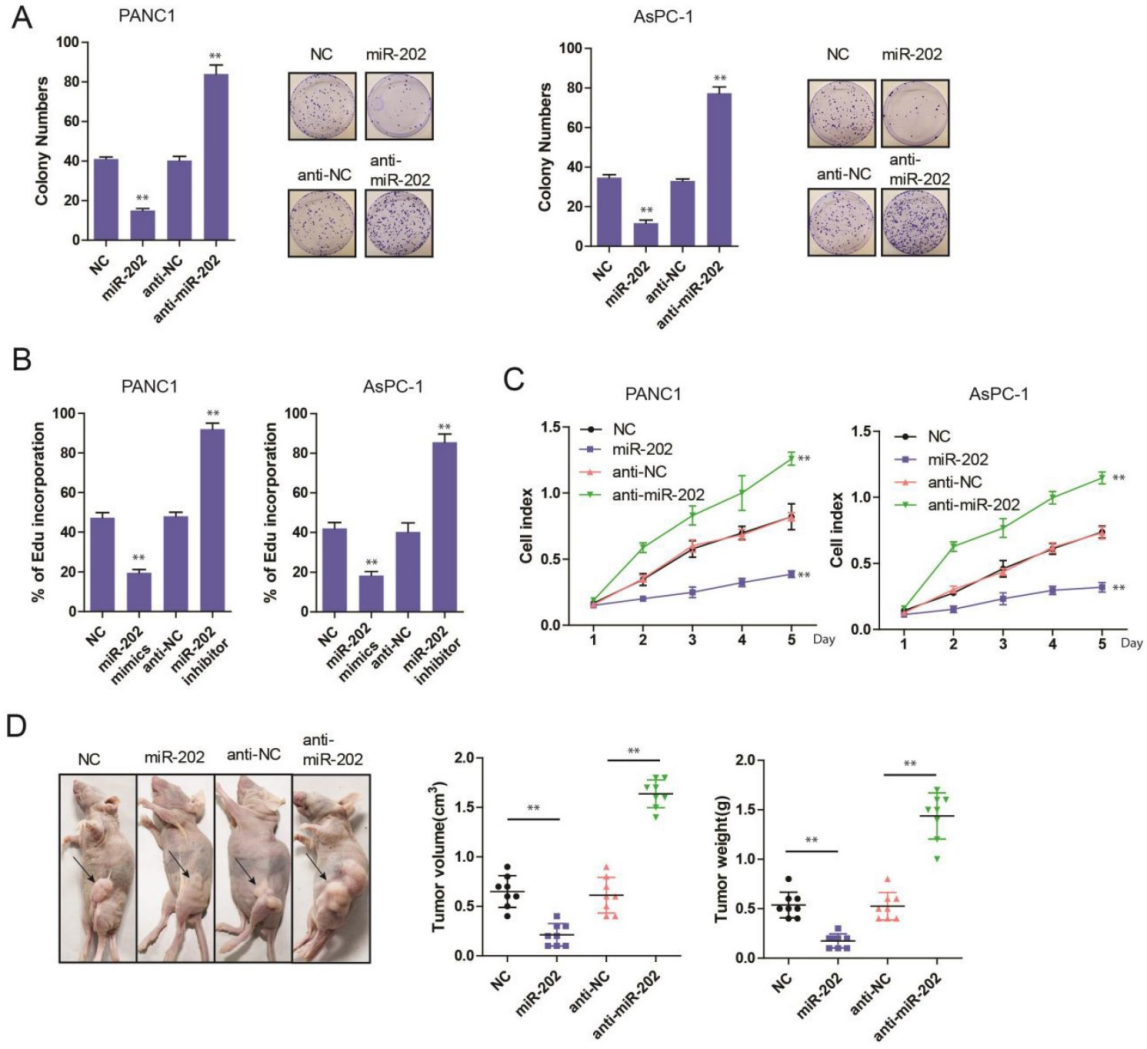
** miR-202 represses PC cell growth and tumorigenicity *in vitro* and *in vivo*.** (A) Colony formation assays of miR-202 overexpression and inhibition in PANC-1 and AsPC-1 cells. (B) Edu incorporation assays of miR-202 mimics and inhibitors in PANC-1 and AsPC-1 cells. (C) The CCK-8 assay was performed to evaluate the effects of miR-202 overexpression and inhibition on cell proliferation in PANC-1 and AsPC-1 cells. (D) Xenograft tumors in nude mice to evaluate miR-202 overexpression and inhibition in PANC-1 cells, tumor volume and weight were also calculated(E) (each group 8 mice).

To evaluate the role of miR-202 in PC, the colony formation assay was performed. we found that miR-202 mimics the repressed colony-forming ability of PC cells (Figure 2A). Both Edu incorporation and proliferation assays also indicated that ectopic expression of miR-202 attenuated growth of PC cells (Figure 2B and C). Conversely, compared with control cells, treatment with miR-202 inhibitor augmented cell growth (Figure 2A, B and C). To investigate whether miR-202 inhibited PC tumorigenic ability *in vivo,* we performed subcutaneous injection on the dorsal flank of mice with stable cells. Consistent with the findings *in vitro*, miR-202 remarkably reduced the tumorigenic ability of PC cells *in vivo* (Figure 2D)*.* As a result, tumor in miR-202 overexpressed group showed a lower weight (Figure 2E). Compared with the control group, tumor volume and weight were dramatically increased in the anti-miR-202 group (Figure 2D and 2E). Together, our data suggest that miR-202 impairs proliferation of PC both *in vitro* and* in vivo.*

### miR-202 inhibits glycolysis in PC

**Figure 3:**
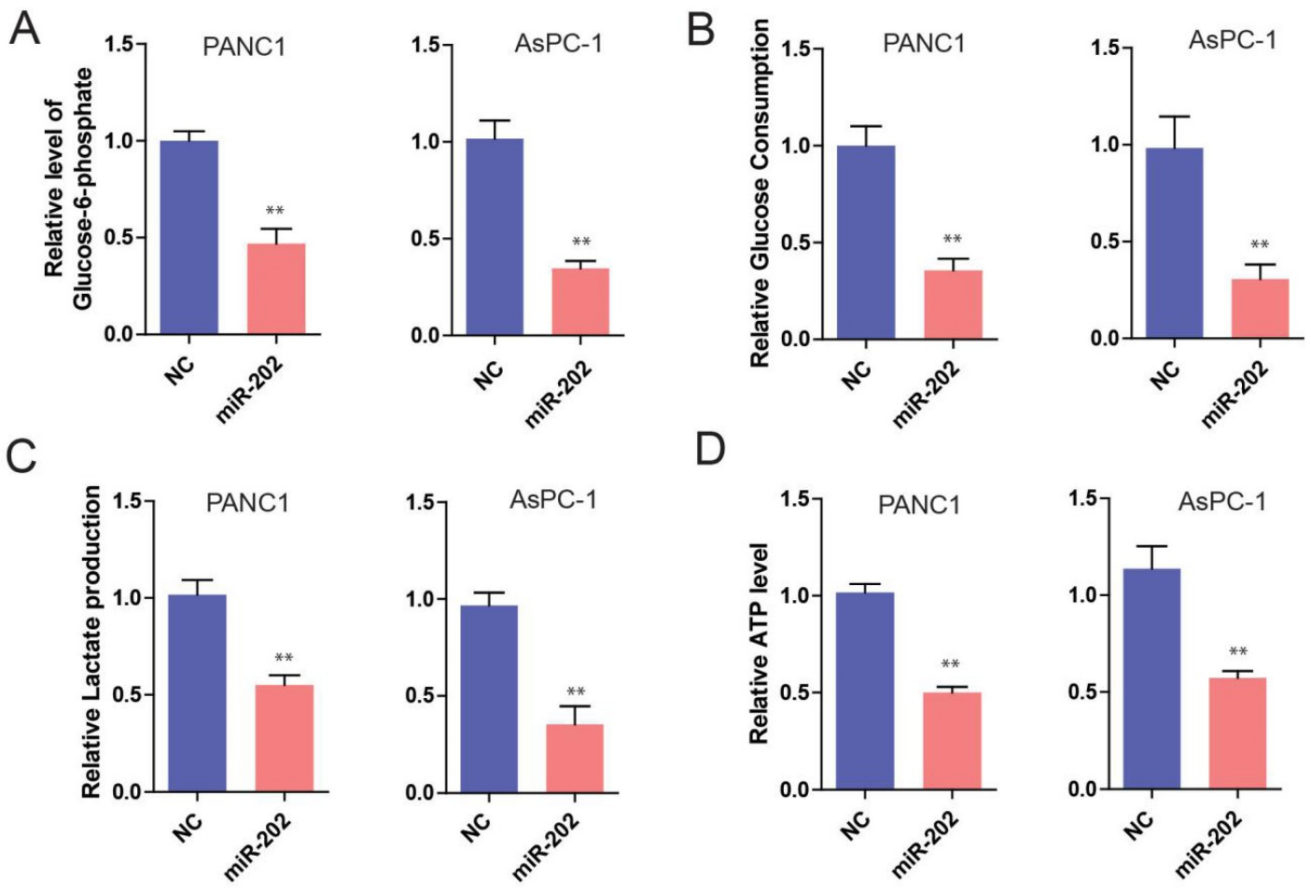
** miR-202 impairs glycolysis in PC cells.** Measurements of glucose-6-phosphate(A), glucose uptake(B), lactate production(C) and ATP level(D) in miR-202 overexpression and control PANC-1 and AsPC-1 cells.

The previous study has demonstrated that tumor cells are inclined to use aerobic glycolysis to obtain enough energy for tumor growth 14. Therefore, cancer cells exhibited a high glycolytic rate with the production of lactate. Recently, substantial microRNAs have been reported in the regulation of cancer cell metabolism, especially glucose metabolism 15. So, we hypothesize that miR-202 might get involved in the regulation of glucose metabolism of PC cells. To test this idea, we measured glucose and glucose metabolites from miR-202 PC cells. It was found that the ectopic expression of miR-202 in PC cells significantly decreased the level of glucose-6-phosphate (G6P), a product of glycogenolysis (Figure 3A). In addition, we also detected a reduction of glucose uptake, lactated production and ATP generation in miR-202 expressed cells (Figure 3B, 3C and 3D). Conversely, silencing the expression of miR-202 facilitated the level of G6P, glucose uptake, lactate production and ATP (data not shown). Collectively, our data suggest that miR-202 can reprogram the glycolysis in PC.

### Hexokinase 2 is a direct target of miR-202 in PC

**Figure 4:**
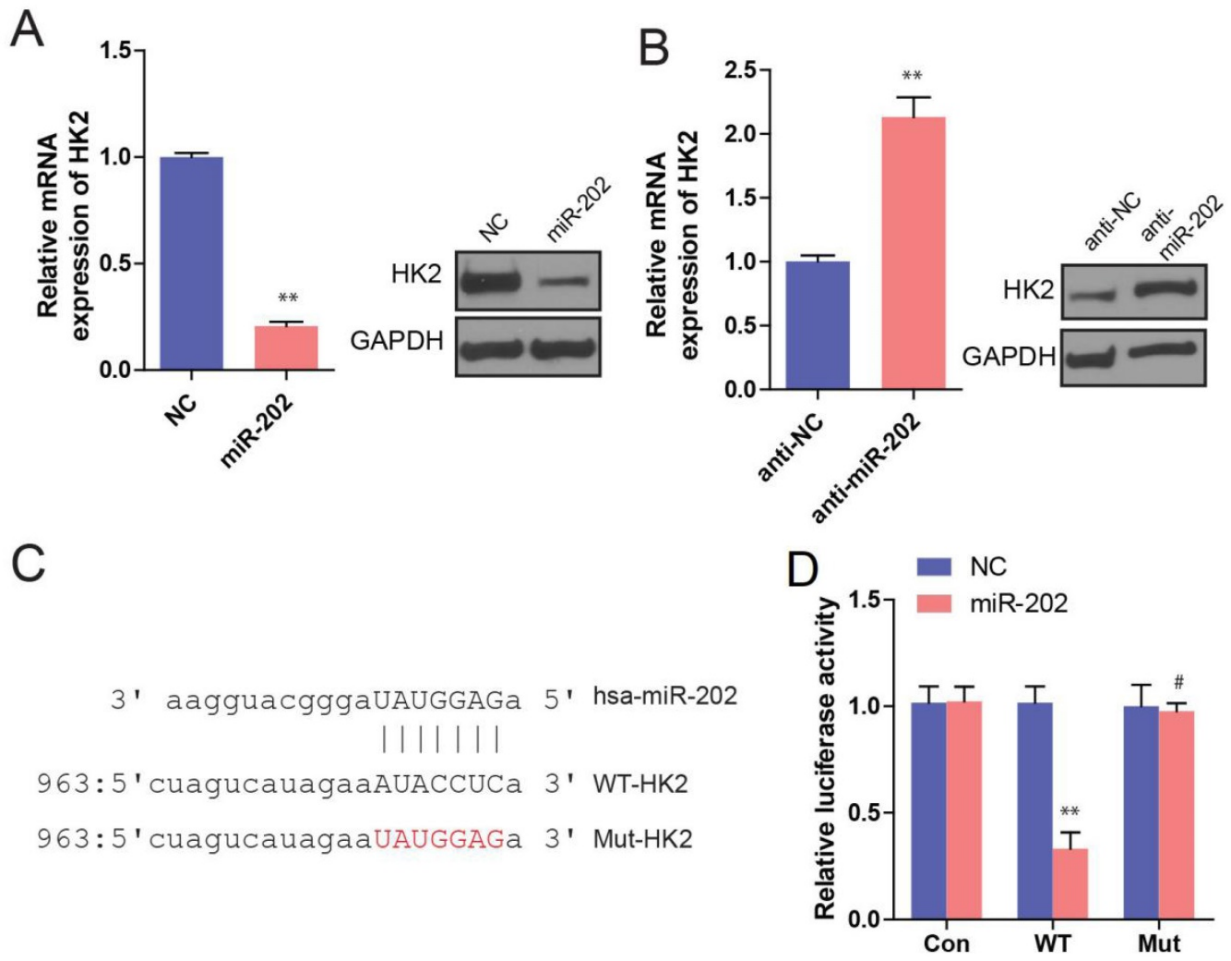
** HK2 is a direct target of miR-202.** (A) Real-time PCR and WB analysis of HK2 mRNA and protein level in PANC-1 cells infected with miR-202-expressing or control vector. (B) Real-time PCR and WB analysis of HK2 mRNA and protein level in PANC-1 cells infected with anti-miR-202-expressing or control vector. (C) the matched sequence of miR-202 and HK2 3'UTR, (D)Luciferase reporter assay for HK2 WT and Mut 3′ UTR in PANC-1 cells.

To understand the mechanisms by which miR-202 inhibits PC cell proliferation, some databases were used to identify miR-202 targets in humans. As miR-202 has been proved to affect glycolysis, we hypothesized that its potential targets should participate in glucose metabolism process. Among those potential targets, HK2 is of particular interest because HK2 is a key enzyme in glucose metabolism, which catalyzes the irreversible rate-limiting step in the glycolytic pathway by phosphorylating G6P in an ATP dependent manner 16. Therefore, we sought to perform a series of experiments to determine whether HK2 is a direct target of miR-202. First, we investigated the effect of miR-202 on HK2 expression. We found that miR-202 overexpression obviously reduced both HK2 mRNA and protein levels in PC cells (Figure 4A). In Contrast, miR-202 silencing caused a dramatic increase in HK2 mRNA and protein levels in PC cells (Figure 4B). Next, we generated wild-type and mutant HK2-3′-UTR expression plasmids that were constructed to a luciferase reporter based on the matched sequence between HK2-3′-UTR and miR-202 (Figure 4C). We found that miR-202 overexpression significantly inhibited luciferase activity of wild-type but not mutant reporter genes in PC PANC-1 cells (Figure 4D).

### HK2 abrogates miR-202 mediated tumor growth and glycolysis inhibition

**Figure 5:**
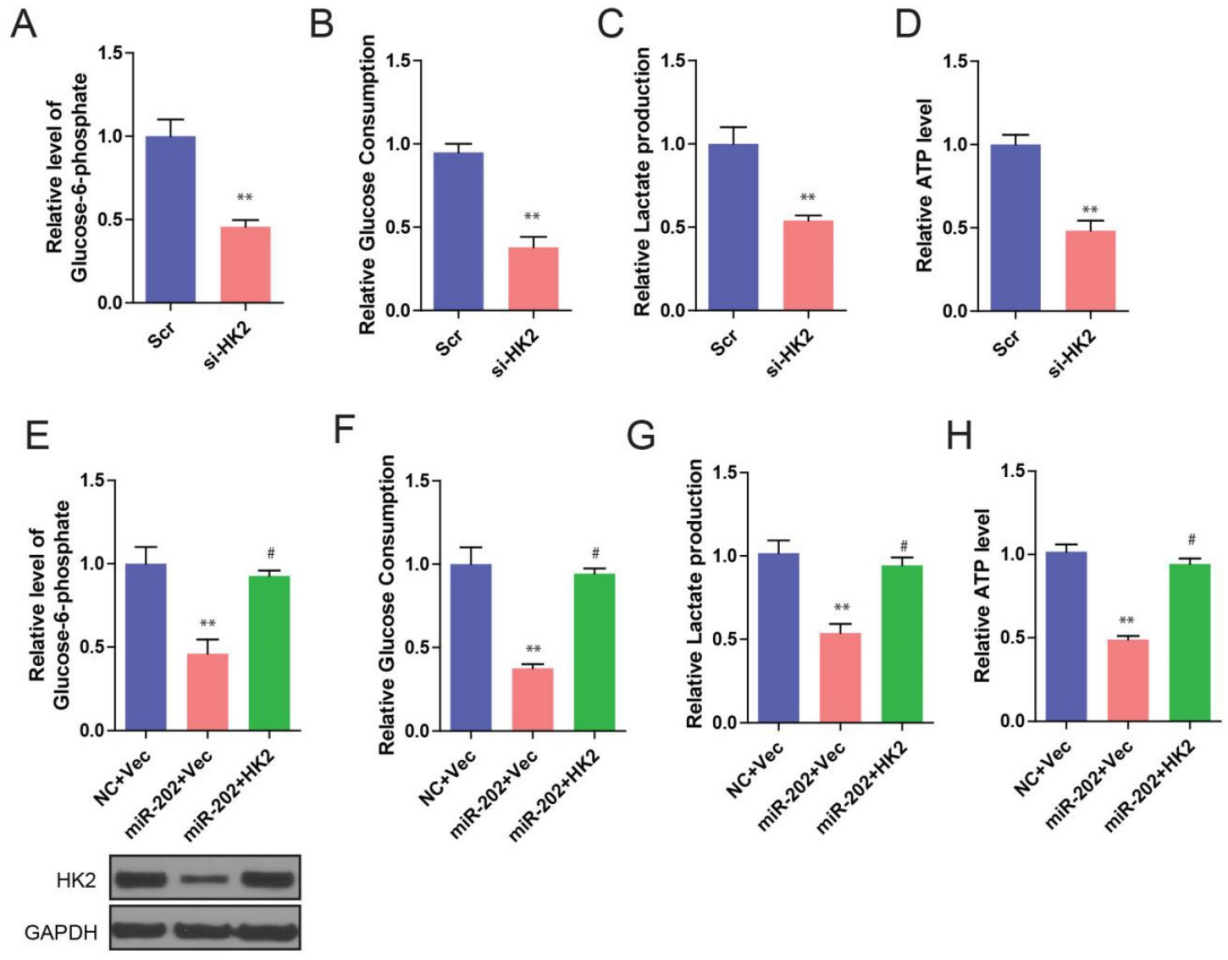
** miR-202 overexpression and HK2 inhibition produce similar changes in glycolysis, which are restored by HK2 ectopic expression.** Measurements of glucose-6-phosphate (A), glucose uptake (B), lactate production (C) and ATP level (D) in HK2 knockdown and control PANC-1 cells. Re-introduction of HK2 in control and miR-202 expression cells, and quantification of glucose-6-phosphate (E), glucose uptake (F), lactate production (G) and ATP level (H) in indicated PANC-1 cells.

We further found that knockdown of HK2 produced similar changes in glycolysis repression to that of miR-202 overexpression (Figure 5A, 5B, 5C and 5D). To determine whether these effects were specifically mediated by HK2 suppression, we utilized an expression construct that encoded the whole HK2 coding sequence within the 3′UTR. As expected, the re-expression of HK2 completely inhibited miR-202-mediated glycolysis inhibition (Figure 5E, 5F, 5G, and 5H). Our data suggest that, after miR-202 overexpression, a decrease in HK2 is required for cells to show decreased glycolytic rate.

**Figure 6:**
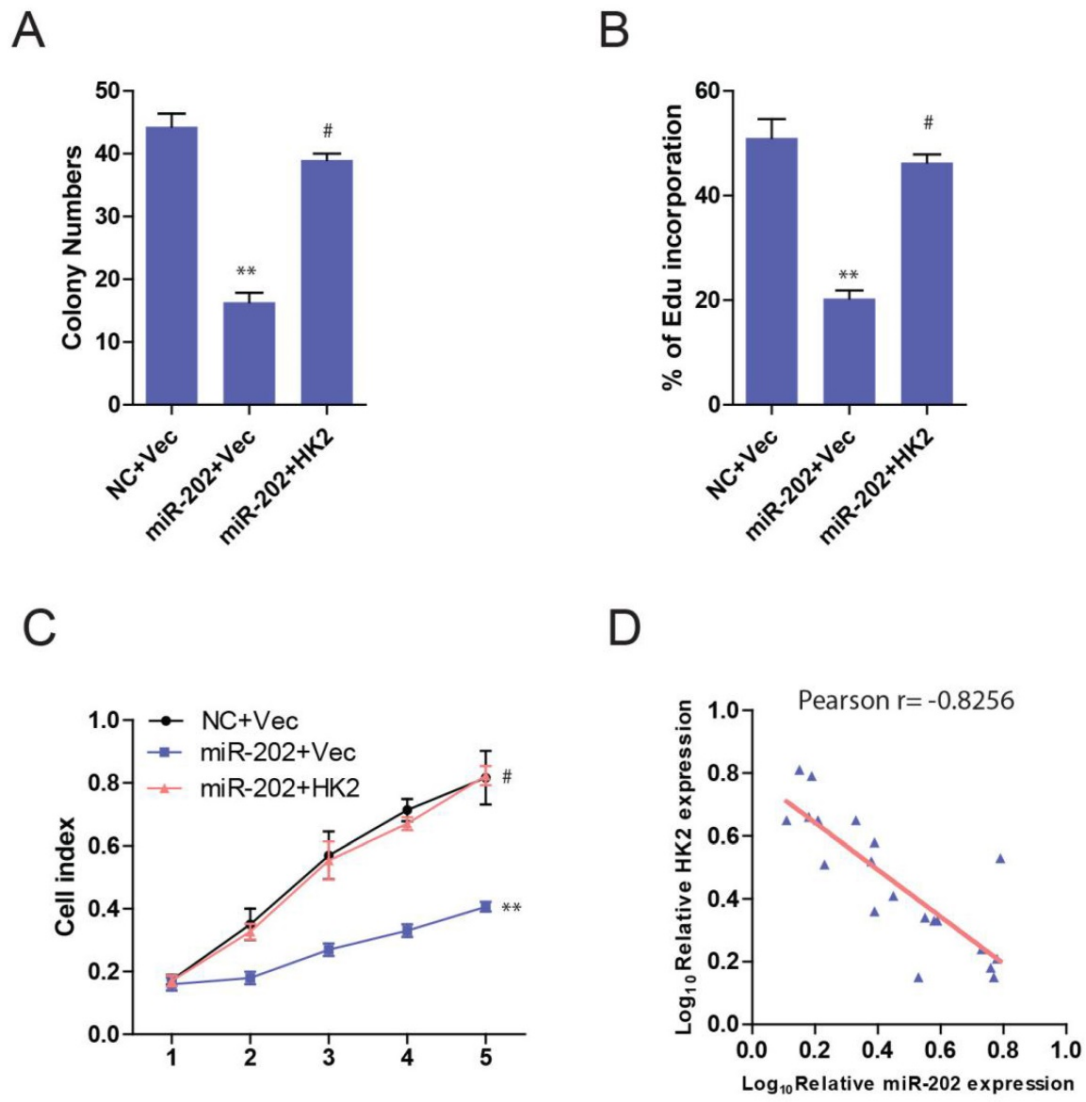
** miR-202 impairs proliferation in PC cells via HK2.** Re-introduction of HK2 in control and miR-202 expression PANC-1 cells, and colony formation assays (A), Edu incorporation (B) and CCK8 assay (C) of indicated cells. (D) Correlation between miR-202 expression and HK2 expression in the clinical samples.

Then we ask whether HK2 connects miR-202 and PC proliferation. Unsurprisingly, PANC-1-miR-202 cells regained the proliferating ability after re-introduction of HK2 (Figure 6A, 6B and 6C). We next examined the clinical relevance of altered miR-202 and HK2 expression in human PC. As the result indicated, the level of miR-202 was inversely correlated with those of the HK2 mRNA level (Figure 6D).

Taken together, these data revealed that the ability of miR-202 to inhibit PC carcinogenesis is attributable, in significant part, to its capacity to inhibit HK2.

## Discussion

Mounting evidences have indicated that miRNAs play a critical role in the process of carcinogenesis. In our present study, we found that the expression of miR-202 was decreased in PC cells and tissues compared with normal controls, ectopic miR-202 expression in PC cells inhibited tumor growth both *in vitro* and *in vivo*. The tumor repressive role of miR-202 is linked to its ability to directly target 3'-UTR of HK2. Furthermore, HK2 overexpression is sufficient to revert PC cell growth inhibition by miR-202. The inverse correlation between miR-202 and HK2 expression is consistent with our data that miR-202 attenuated PC carcinogenesis by diminishing HK2 expression. These findings have implicated the potential application of miR-202/HK2 pathway in PC treatment.

Reprogramming energy metabolism has emerged as a distinguishing feature of cancer. It affects multiple metabolic processes including nucleic acid synthesis, amino acid metabolism, lipid synthesis, glycolysis, tricarboxylic acid cycle, fatty acid metabolism and NAD metabolism 17,18. Compared with the non-transformed cells, the cancer cells display special metabolic phenotypes such as enhanced glucose uptake, elevated lactate production even under normoxia, to facilitate cell growth and division. The phenomenon was termed as the Warburg effect 19. Thus, intervening the metabolic process might be a promising strategy for target cancer therapy. In recent years, growing evidence shows that non-coding RNAs function as crucial regulators of cancer metabolism. Specifically, miRNA is extensively investigated in the control of the manifold metabolic process through intricate mechanisms, including directly targeting transporters of metabolic processes or critical metabolic enzymes and regulating transcription factors, oncogenes/tumor suppressors as well as multiple oncogenic signaling pathways 20. Recent findings uncovered that several miRNAs, such as miR-23a/b, miR-199a, miR-138, participated in gluconeogenesis and glycolysis 21-23. In the present study, we report that miR-202 is an important miRNA linking to PC cell metabolism. Furthermore, our study demonstrated that miR-202 repressed glycolysis and lactate production in PC cells. Although previous studies reported the role of miR-202 was controversial. Our study adds another evidence that miR-202 can act as a tumor suppressor.

Cancer glucose metabolism is a multistep process catalyzed by a few critical rate-limiting enzymes. HK 2 is a pivotal enzyme, which catalyzes the first irreversible rate-limiting step in the glycolytic pathway where glucose is phosphorylated to G6P with concomitant de-phosphorylation of ATP 16. There are four major isoforms of HK family encoded by separate genes: HK1, HK2, HK3, and HK4 (also known as glucokinase) 24. HK1 is ubiquitously expressed among all mammalian tissues, while HK2 is expressed in adipose, skeletal, and cardiac muscles, which are all insulin-sensitive. Low levels of HK3 are detected in human tissues and HK4 expression is limited to the pancreas and liver 25. Notably, HK2, and it is distinctly elevated in various cancers and contributes to aerobic glycolysis. Previous studies have shown that HK2 was upregulated by in breast, lung, liver, and colon cancers, which was targeted by miR-143^26^ and miR-199a-5p 22. Therefore, it is having been proposed as a potential therapeutic target for cancers. In this study, we demonstrated that HK2 is directly targeted by miR-202 in PC cells. Additionally, Moreover, our data demonstrate that HK2 is essential for glucose metabolism of PC cells and plays a key role in PC cell proliferation. Thus, our data reveal a novel mechanism for HK2 regulation by miRNAs in PC.

In conclusion, our study demonstrated that miR-202 repressed PC cell proliferation, both *in vitro* and *in vivo* via inhibition of HK2 expression, leading to rate-limiting glycolysis enzymes' inactivation. miR-202 abundance is negatively correlated with HK2 expression, which predicts PC patient prognosis. Our findings outline the importance of the miR-202/HK2 axis in Warburg effect and PC carcinogenesis, highlighting the potential role of miR-202 in a therapeutic application for PC.

## Figures and Tables

**Table 1:** Relationship between miR-202 expression and clinicopathologic features of PC Patients (n=101)

Features		Relative miR-202 expression		*P* Value
		High(n=50)	Low(n=51)	
Gender	Male	23	25	NS
	Female	27	26	
Age	≤ 65	20	22	NS
	> 65	30	29	
CA199 (μg/L)	≤ 100	11	8	NS
	> 100	39	43	
TNM stage	I-IIA	40	15	NS
	IIB-IV	10	36	
Pathological grade	Well-moderate	33	23	NS
	Poor	17	28	
Tumor size(cm)	≤ 2.5	35	17	<0.0001
	> 2.5	15	34	
Vascular invasion	Yes	21	27	NS
	No	29	24	
Lymph node metastasis	Yes	11	38	<0.0001
	No	39	13	

Note: PDAC patients were divided into miR-202 'High' group (Relative fold change was higher than the median) and 'Low' group (Relative fold change was lower than the median). Abbreviations: CA199, carbohydrate antigen 19-9; TNM, tumor-nodes-metastasis; NS, not significant between any groups. Differences among variables were assessed by χ^2^ or Fisher's exact χ^2^ test.

**Table 2:** Univariate and multivariate analyses of factors associated with survival in PC patients

Features	Univariate Analysis			Multivariate Analysis		
	HR	95% CI	P value	HR	95% CI	P value
**Gender**						
Male vs. Female	0.898	0.548-1.472	*N.S.*			
**Age**						
> 65 vs. ≤ 65	1.451	0.891-2.361	*N.S.*			
**CA199 (μg/L)**						
> 100 vs. ≤ 100	1.962	1.095-3.515	**0.024**			
**TNM stage**						
I-IIA vs. IIB-IV	0.438	0.243-0.788	**0.006**	0.673	0.446-0.889	**0.034**
**Pathological grade**						
Well-moderate vs. Poor	0.475	0.278-0.813	**0.007**			
**Tumor size(cm)**						
≤ 2.5 vs. > 2.5	0.368	0.226-0.769	**0.002**	0.458	0.352-0.816	**0.012**
**Vascular invasion**						
Yes vs. No	1.979	1.050-3.729	**0.035**			
**Lymph node metastasis**						
Yes vs. No	1.917	1.138-3.232	**0.015**			
**miR-202 expression**						
Low vs. High	2.205	1.302-3.731	**0.003**	1.850	1.064-3.217	**0.029**
